# Rabies Virus Infection Causes Pyroptosis of Neuronal Cells

**DOI:** 10.3390/ijms25115616

**Published:** 2024-05-22

**Authors:** Dongling Yu, Rong Jin, Jundan Liu, Chuanliang Zhang, Chenxing Duan, Xi Luo, Wenhao Yang, Cheng Liu, Jingjing Liang, Xiaoning Li, Tingrong Luo

**Affiliations:** College of Animal Science and Veterinary Medicine, Guangxi University, Nanning 530004, China; yudongling@st.gxu.edu.cn (D.Y.); jinrong@st.gxu.edu.cn (R.J.); liujundan@st.gxu.edu.cn (J.L.); zhangchuanliang@st.gxu.edu.cn (C.Z.); 2118302006@st.gxu.edu.cn (C.D.); 2218302023@st.gxu.edu.cn (X.L.); 2218302038@st.gxu.edu.cn (W.Y.); liucheng0621@gxu.edu.cn (C.L.); jiliang@upenn.edu (J.L.)

**Keywords:** rabies virus (RABV), neuronal cell, transcriptomics profile, pyroptosis, Gsdmd

## Abstract

Rabies virus (RABV) is a neurotropic virus that causes fatal neurological disease, raising serious public health issues and attracting extensive attention in society. To elucidate the molecular mechanism of RABV-induced neuronal damage, we used hematoxylin–eosin staining, transmission electron microscopy, transcriptomics analysis, and immune response factor testing to investigate RABV-infected neurons. We successfully isolated the neurons from murine brains. The specificity of the isolated neurons was identified by a monoclonal antibody, and the viability of the neurons was 83.53–95.0%. We confirmed that RABV infection induced serious damage to the neurons according to histochemistry and transmission electron microscope (TEM) scanning. In addition, the transcriptomics analysis suggested that multiple genes related to the pyroptosis pathway were significantly upregulated, including *gasdermin D* (*Gsdmd*), *Nlrp3*, *caspase-1*, and *IL-1β*, as well as the chemokine genes *Ccl2*, *Ccl3*, *Ccl4*, *Ccl5*, *Ccl7*, *Ccl12*, and *Cxcl10*. We next verified this finding in the brains of mice infected with the rRC-HL, GX074, and challenge virus standard strain-24 (CVS-24) strains of RABV. Importantly, we found that the expression level of the Gsdmd protein was significantly upregulated in the neurons infected with different RABV strains and ranged from 691.1 to 5764.96 pg/mL, while the basal level of mock-infected neurons was less than 100 pg/mL. Taken together, our findings suggest that Gsdmd-induced pyroptosis is involved in the neuron damage caused by RABV infection.

## 1. Introduction

Rabies is an acute fatal zoonosis that affects the central nervous system (CNS) of mammals, including humans and animals, with a 100% mortality rate [1,2]. Its causative pathogen, rabies virus (RABV), belongs to the genus *Lyssavirus* of the family Rhabdoviridae. RABV consists of a single-stranded, negative-sense RNA genome estimated at 12 nucleotides (nt) that encodes five structural proteins in the following order: nucleoprotein (N), phosphoprotein (P), matrix protein (M), glycoprotein (G), and RNA-dependent RNA polymerase (L) [3]. According to the World Health Organization (WHO) statistics, rabies causes approximately 60,000 human deaths worldwide annually [1,4]. Domestic dogs are the main source of human infection [5]. Owing to the limited medical resources and vaccines in developing countries, human rabies cases mainly occur in Africa and Asia [6]. No effective approach is available for rabies therapy once clinical symptoms occur [7]. Although the pathogenesis of rabies virus has been extensively studied, including functional determinants [8,9,10,11], innate immune response [12,13], and neuron death [4,14], currently, the pathogenic mechanism by which RABV causes host disease is unclear. The host immune system can recognize viruses as foreign matter and activate signal transduction pathways to clear them. However, RABV is a neurotropic microorganism that invades the nervous system and causes pathological changes in the CNS. Although specific innate immune sentinel cells such as microglia exist in the CNS, which can secrete cytokines and/or recruit the peripheral immune cells into the local inflammatory area to clear the virus [15], the neurons in the CNS are still damaged during RABV infection.

In recent years, studies have found that the recognition of RABV by resident immune sentinel cells (such as microglial cells), as well as by immune cells infiltrating into the CNS, might lead to a chemotactic environment and cause inflammation in the infected brain [15,16]. Nevertheless, on the one hand, the acute inflammation participates in viral inhibition and clearance, and on the other hand, the excessive inflammation leads to tissue damage [17].

Some studies have demonstrated that murine models of RABV infection induced different signaling pathways of innate immune response and programmed cell death [18]. Pyroptosis is a type of regulated cell death mediated by gasdermin [19,20]. The analysis of murine brain transcriptomes after experimental Duvenhage lyssavirus (a rabies-related virus from Africa) infection showed activation of the immune response and pyroptotic cell death pathways [4]. Our research found that RABV infection might cause neuron damage. However, we cannot be certain whether or not pyroptosis takes part in the process of neuron damage. In this study, we aimed to explore the mechanism of damage to neuronal cells in order to clarify the relationship between neuronal damage and pyroptosis.

## 2. Results

### 2.1. Pathogenicity of RABV in Mice

**Figure 1:**
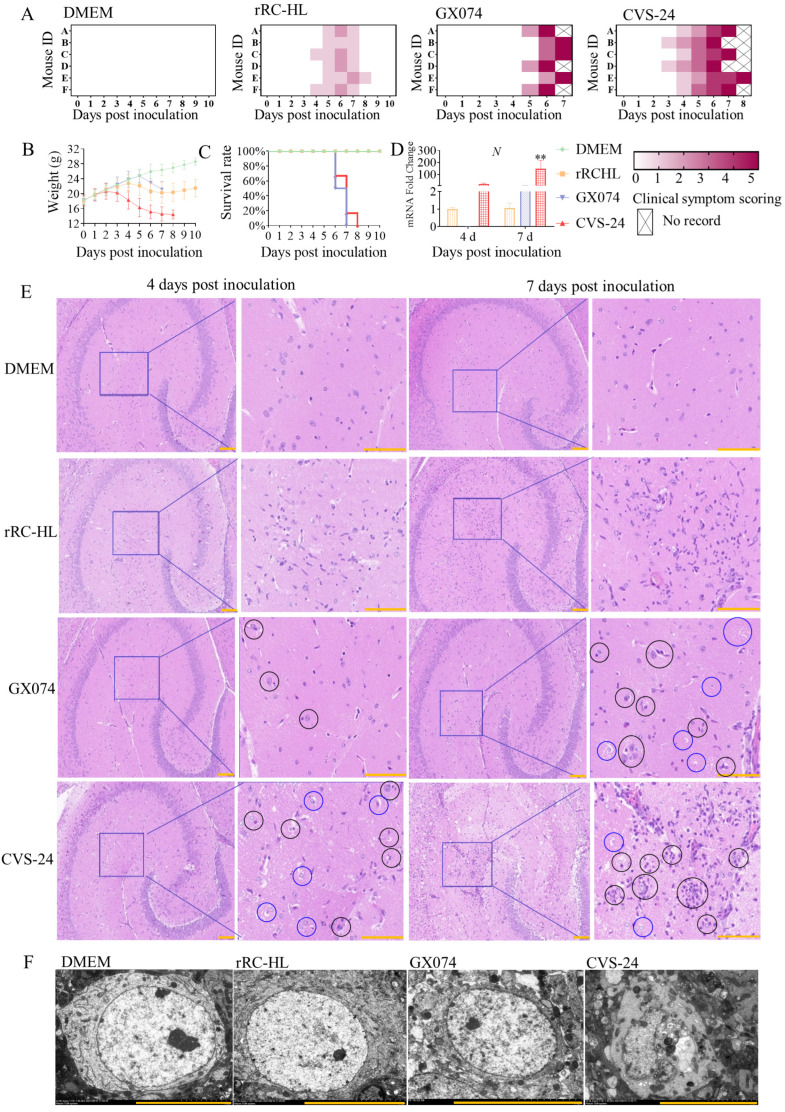
Rabies virus infection caused damage and death of neuronal cells in the murine brain: (**A**) Clinical scores of mice infected with rabies virus (RABV). (**B**) Clinical weight changes in mice infected with rabies virus (4 weeks old). (**C**) Percentage survival (%) of mice infected with rabies virus. (**D**) Fold changes in the RABV *N* gene in the brains of mice infected with RABV. (**E**) HE staining of brain sections in the hippocampi of mice infected with RABV (blue circles: damage or death of neuronal cells; black circles: neuronophagia and microglial nodules, Scale bar = 100 μm). (**F**) Transmission electron microscope images of the neuronal cells in the hippocampus (Scale bar = 100 μm). Asterisks indicate the statistical significance as follows: **, *p* < 0.01.

To investigate the pathological changes in brain tissue infected with RABV in vivo, 4-week-old Kunming (KM) mice were infected with the rRC-HL, GX074, or CVS-24 strains of RABV via the intracerebral (i.c.) route. The mice infected with rRC-HL developed mild symptoms including rough fur and transient weight loss from 4 days post inoculation (dpi), and then they recovered at 9 dpi. The clinical score of the rRC-HL group was judged to be 2.0. The clinical symptoms of the mice infected with the standard challenge strain CVS-24 began at 3 dpi, including weight loss, rough fur, mental depression, and paralysis of the fore or hind limbs, and they then progressed to death at 6 dpi. The clinical score of the CVS-24 group was determined to be 4.0. The mice infected with the street strain GX074 began to show weight loss, coarse hair, and sensitivity to stimulation at 5 dpi. Fifty percent of the mice infected with the GX074 strain presented typical neurological symptoms such as paralysis of the fore or hind limbs, and then they rapidly progressed to severe seizures and died at 6 dpi (Figure 1A–C). The brains of the mice infected with RABV were used to detect the viral *N* gene. All of the brain samples were RABV-positive (Figure 1D). 

The brain samples were embedded in paraffin, and the brain tissue sections were prepared to observe pathological changes via HE staining. As shown in Figure 1E, no obvious pathological changes were seen in the tissue sections from the murine brains injected with Dulbecco’s modified Eagle’s medium (DMEM) at 4 and 7 dpi. The brain tissues showed clear nuclear membranes of neurons, light nuclei, and loose chromatin in the hippocampal region. In the murine brains infected with rRC-HL at 4 and 7 dpi, small amounts of inflammatory cells (including neuroglial cells) were found to be infiltrating the hippocampal region. The murine brains infected with CVS-24 had inflammatory cells infiltrating the hippocampal region, as well as the proliferation of neuroglial cells, which surrounded the neurons (i.e., neuronophagia), and they showed neuronal damage or neuron death at 4 and 7 dpi. Only a few pathological lesions were observed in the murine brain tissue infected with GX074 at 4 dpi; however, many dead neurons were observed at 7 dpi. The pathological changes caused in these brain tissues by the different viruses were consistent with their respective clinical symptoms.

The ultrastructural changes in neurons were evaluated by means of TEM observation. As shown in Figure 1F, the neurons in the DMEM group presented a large and round morphology with an oval nucleus and an intact nuclear membrane. The nucleus was not obviously changed in the rRC-HL infection group. Meanwhile, in the GX074 group, the nuclear membrane became rough, the nucleus presented a concentrated state and became smaller, and vesicles appeared in the cytoplasm around the nucleus. In the CVS-24-infected group, the nuclear membrane was partially dissolved and broken; the nuclei were irregular, the organelle structure was disordered and disintegrated, and vacuoles were formed.

### 2.2. Analysis of Differentially Expressed Genes (DEGs) Based on the Transcriptome Expression Profile

**Figure 2:**
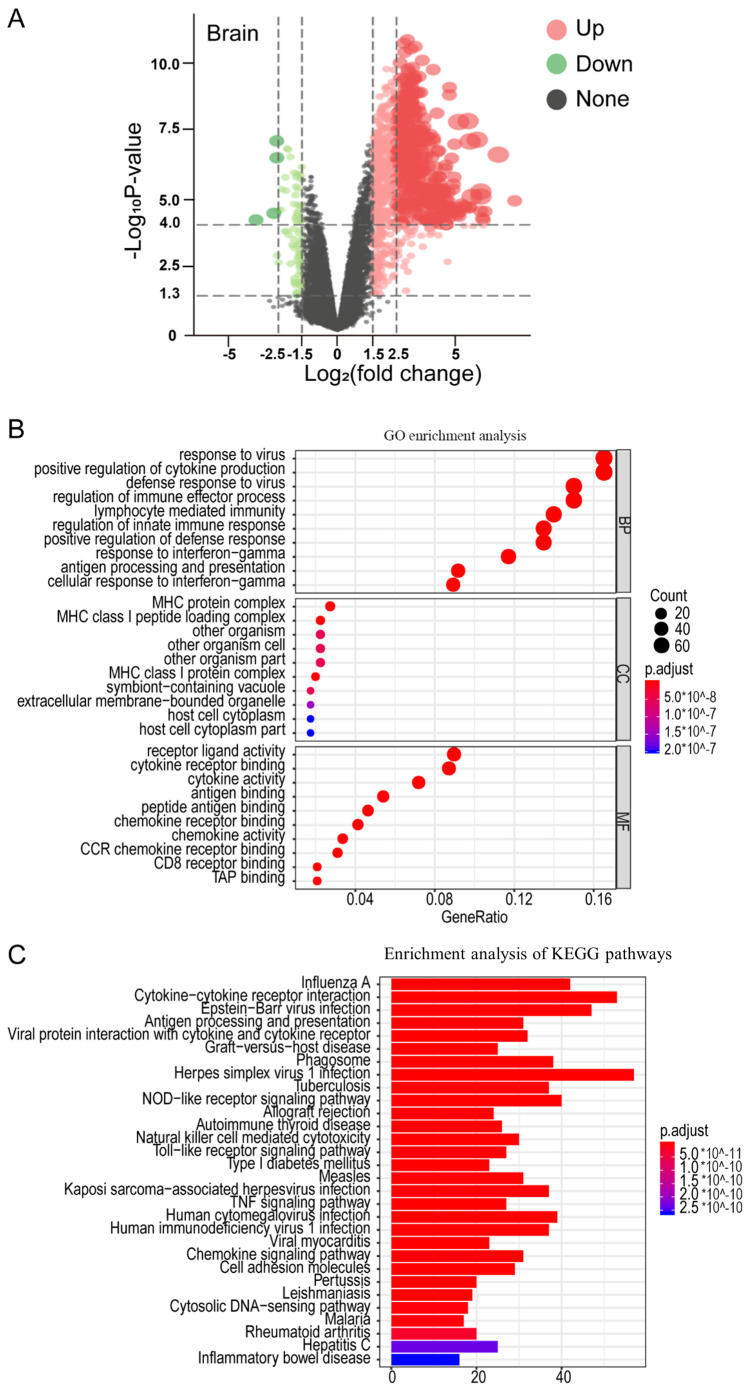
The analysis of differentially expressed genes (DEGs) and the analysis of Gene Ontology (GO) and KEGG pathways in the brains of mice infected with RABV: (**A**) Volcano plot of the DEGs in the brains of mice infected with RABV. The color, from green to red, shows the progression from low to high expression levels, respectively. (**B**,**C**) The analysis of Gene Ontology (GO) and KEGG signaling pathways in the brains of mice infected with RABV.

To understand the mechanism by which RABV causes pathological changes in the brains of mice, the transcriptome expression profile of mice infected with the CVS strain of RABV was analyzed (NCBI GEO database, GSE30577). The analysis of the transcriptome expression profile revealed 548 DEGs in the brains of the mice infected with the virulent RABV CVS strain, i.e., 532 upregulated and 16 downregulated genes (Figure 2A). The 532 upregulated DEGs contained various cytokines, such as interferons, interleukins, TNF, and chemokines, most of which are related to immune inflammation. Furthermore, it was found that several DEGs associated with pyroptosis were upregulated, including *gasdermin D* (*Gsdmd*), *Nlrp3*, *caspase-1* (*Casp1*), and *IL-1β*, as well as a few chemokine genes (*Ccl2*, *Ccl3*, *Ccl4*, *Ccl5*, *Ccl7*, *Ccl12*, *Cxcl10*, etc.) (Table 1). All the DEGs were used for further bioinformatics analysis to explore the pathogenic mechanism of RABV.

The DEGs were mapped to Gene Ontology (GO) for functional annotation of the modulated genes, and 1225 Gene Ontology (GO) items were found in the brains of the mice infected with the virulent RABV CVS strain. The results showed that the DEGs in the brains of the RABV-infected mice were related to biological processes (BPs), which were associated with the immune response, such as response to the virus (77 genes), defense response to the virus (76 genes), regulation of immune effector process (76 genes), and regulation of innate immune response (62 genes). Meanwhile, it was found that Gsdmd is involved in the positive regulation of cytokine production, the defense response to the bacterium, cytokine secretion, pyroptosis, and other related processes. The dominant subcategories of the cellular component (CC) included 12 genes related to “MHC class I protein complex”, 10 genes related to “MHC class I peptide loading complex”, and some genes relating to “other organism” and “host cell cytoplasm”, among others. The dominant subcategories of molecular function (MF) included receptor ligand activity (44 genes), cytokine receptor binding (41 genes), cytokine activity (37 genes), and some genes relating to “chemokine receptor binding” and “chemokine activity” (Figure 2B). 

The KEGG pathway enrichment analysis revealed 66 signal transcription pathways in the brains of the mice infected with the CVS strain. According to the KEGG pathway enrichment analysis, these signal transcription pathways were related to “cytokine-cytokine receptor interaction”, “NOD-like receptor signaling pathway”, “Toll-like receptor signaling pathway”, “TNF signaling pathway”, and “Chemokine signaling pathway”. It was found that the signaling pathways activated by RABV infection were similar to those induced by influenza A, Epstein–Barr virus infection, herpes simplex virus 1 infection, human cytomegalovirus infection, and hepatitis C infection, among others (Figure 2C).

### 2.3. Analysis of Protein–Protein Interaction (PPI) Based on the Transcriptome Profile

**Figure 3:**
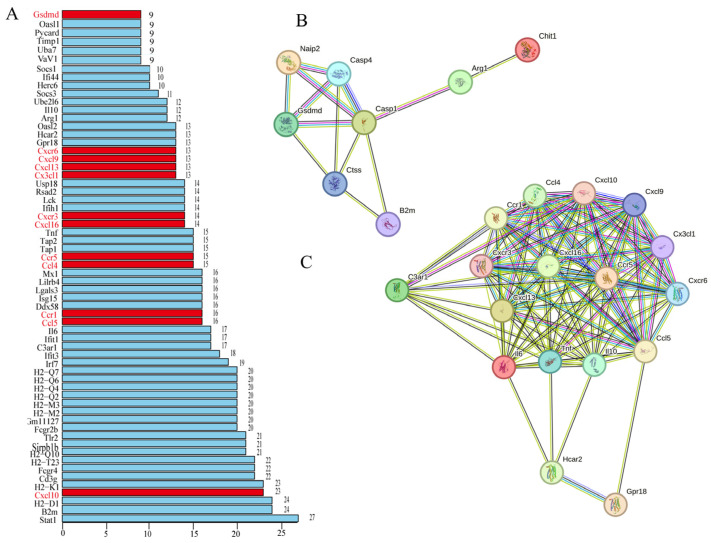
Protein–protein interaction (PPI) network: (**A**) The 62 proteins that could interact with at least 9 proteins were identified as the hub proteins based on the PPI network (The proteins of interest is highlighted in red). (**B**) The PPI networks of the Gsdmd signaling pathway. (**C**) The PPI networks of Tnf, IL-6, IL-10, and chemokines.

A PPI network was constructed and used to identify the specific interacting proteins in the brains of the mice infected with RABV in order to develop an in-depth understanding of the correlation. Genes that were enriched in the previous analyses were extracted using STRING analysis, and the network was visualized using the Cytoscape software (version 3.8.2), with a threshold interaction score of 0.90. The selected proteins were found to interact with at least nine other proteins, including Dsdmd (9), Il-10 (12), TNF (15), Il-6 (17), H2 superfamily (20–24), Cxcl10 (23), B2m (24), and Stat1 (27) (Figure 3A). These proteins are suggested to be pivotal hub proteins in the central nervous system during RABV CVS infection. In particular, the proteins that interacted with Gsdmd included Casp1, Casp4, Naip2, Ctss, B2m, Hpse, Arg1, Chit1, and Ptx3; these protein interactions might be involved in pyroptosis, implying that RABV infection might lead to pyroptosis (Figure 3B). The proteins that interacted with chemokines were cytokine proteins, for instance, Il-6, Il-10, Ccl5, Cxcl10, etc., implying that these PPIs might be involved in the immune inflammation response (Figure 3C).

### 2.4. Validation of DEGs in Murine Brains

**Figure 4:**
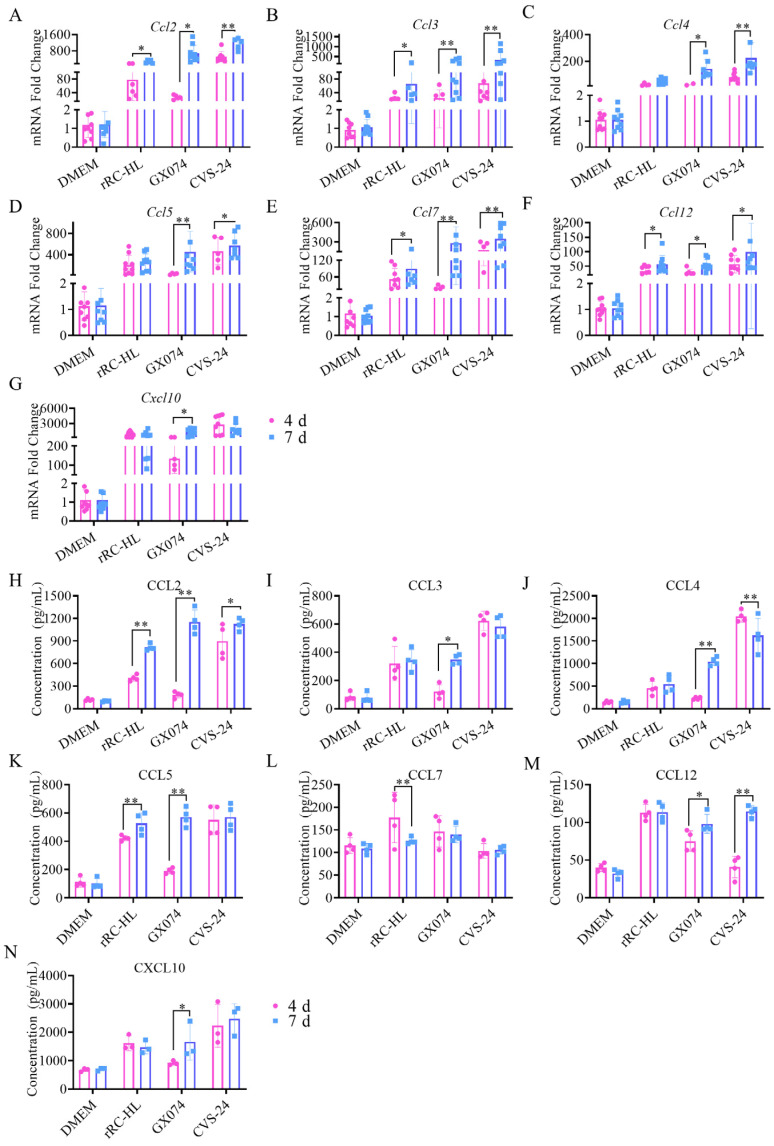
Determination of chemokine levels in the brains of mice infected with RABV. (**A**–**G**) The mRNA expression levels of chemokines in the brains of mice infected with RABV were measured as the mean ± standard deviation (SD) and compared to a specific endogenous housekeeping gene (β-actin gene). (**H**–**N**) Enzyme-linked immunosorbent assay (ELISA) was used to detect the protein expression levels of the chemokines in the brains of all mouse groups. All the statistical significances were determined via Student’s test and one-way ANOVA. Asterisks indicate the statistical significance as follows: *, *p* < 0.05; **, *p* < 0.01.

The results of our analysis of the transcriptome expression profiles imply that there are many chemokines involved in PPI during RABV infection. To validate the authenticity of the transcriptome expression profiles, the mRNA expression levels of seven chemokine genes (*Ccl2*, *Ccl3*, *Ccl4*, *Ccl5*, *Ccl7*, *Ccl12*, and *Cxcl10*) in the brains of mice infected with the rRC-HL, GX074, and CVS-24 strains of RABV were measured at 4 and 7 dpi in vivo. The results showed that the mRNA expression levels of all seven chemokine genes in the brains of the mice infected with rRC-HL, GX074, and CVS-24 had increased noticeably at 4 and 7 dpi (Figure 4A–G), and these data were consistent with the transcriptome expression profiles (Figure 2B, Table 1). Nevertheless, the mRNA expression levels of the four chemokine genes (*Ccl2*, *Ccl5*, *Ccl7*, and *Cxcl10*) in the brains of the mice infected with the GX074 strain at 4 dpi were slightly lower than those at 7 dpi, but they rapidly increased at 7 dpi. The mRNA expression levels of nine chemokine genes in the brains of the CVS-24-infected mice were significantly higher than those in the rRC-HL and GX074 groups at 4 and 7 dpi. The data displayed similar trends to the analysis of the transcriptome expression profiles. Subsequently, the protein levels of seven chemokines from the murine brain samples were measured using enzyme-linked immunosorbent assay (ELISA) kits. The results demonstrated that the protein expression levels of the seven chemokines in the brains of the mice infected with rRC-HL, GX074, and CVS-24 had all increased at 4 and 7 dpi, and their levels at 4 dpi were generally lower than those at 7 dpi. However, the protein levels of the five chemokines CCL2, CCL3, CCL4, CCL5, and CXCL10 in the brains of the mice infected with GX074 at 4 dpi (185.7, 121.3, 230.5, 189.4, and 967.0 pg/mL, respectively) were lower than those at 7 dpi (1154.2, 349.3, 1047.25, 571.4, and 1660.0 pg/mL, respectively). The upward trend of the protein levels was consistent with that of the mRNA levels (Figure 4H–N). 

**Figure 5:**
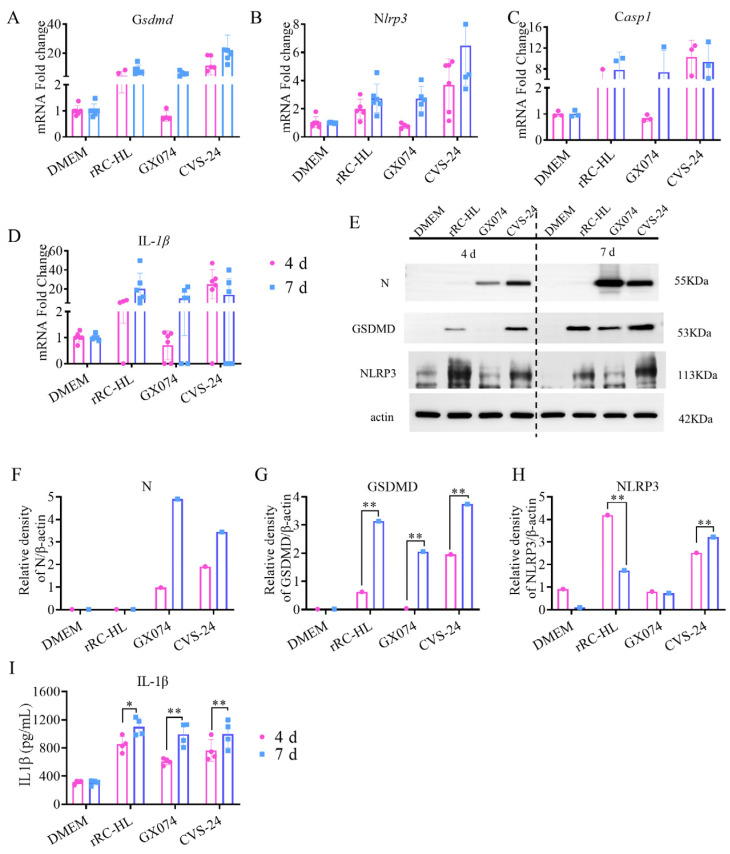
Determination of pyroptosis-related factors in the brains of mice infected with RABV: (**A**–**D**) Determination of Gsdmd, Nlrp3, Casp1, and IL-1β in the brains of mice infected with RT-qPCR. (**E**) Western blot assay was used for detecting the expressions of the RABV N, Gsdmd, and NLRP3 proteins in all groups. (**F**–**H**) Quantification of the RABV N, Gsdmd, and NLRP3 proteins based on the Western blot assay. (**I**) ELISA for detection of the IL-1β protein. Asterisks indicate the statistical significance as follows: *, *p* < 0.05; **, *p* < 0.01.

Based on the transcriptome expression profiles, some genes that were upregulated in the brains of the RABV-infected mice were determined to be related to the pyroptosis pathway (Figure 5A–D). At 4 dpi, the expression levels of the *Gsdmd*, *Nlrp3*, *Casp-1*, and *IL-1β* genes were upregulated in the brains of the mice infected with rRC-HL (by 4.45-, 1.97-, 5.45-, and 5.40-fold, respectively) and CVS-24 (by 11.31-, 3.7-, 10.27-, and 29.83-fold, respectively), whereas the expression levels of these genes were relatively low in the brains of the mice infected with GX074 (upregulated by 0.80-, 0.79-, 0.84-, and 1.06-fold, respectively). At 7 dpi, the expression levels of the *Gsdmd*, *Nlrp3*, *Casp-1*, and *IL-1β* genes were significantly upregulated in the brains of the mice infected with rRC-HL (by 8.57-, 2.74-, 7.82-, and 202.28-fold, respectively), GX074 (by 5.94-, 2.70-, 7.38-, and 15.34-fold), and CVS-24 (by 22.33-, 6.49-, 9.33-, and 27.82-fold, respectively). Similarly, the mRNA expression levels of those four genes in the brains of the mice infected with the GX074 strain at 4 dpi were found to be slightly lower than those at 7 dpi. Western blotting and ELISA were also used to detect the protein expression levels of the factors Gsdmd, Nlrp3, and IL-1β related to the pyroptosis pathway. The results of the Western blot analysis showed that the expression levels of the Gsdmd and NLRP3 proteins were significantly increased in the GX074- and CVS-24-infected groups compared with the DMEM group at 7 dpi (Figure 5E–H). The IL-1β protein levels were found to be equivalent to the mRNA levels, as shown in Figure 5I. The IL-1β protein was significantly upregulated in the brains of the mice infected with rRC-HL (852.11 pg/mL), GX074 (606.67 pg/mL), and CVS-24 (764.92 pg/mL) at 4 dpi. Similarly, the IL-1β protein was significantly upregulated in the brains of the mice infected with rRC-HL (1101.16 pg/mL), GX074 (993.83 pg/mL), and CVS-24 (997.35 pg/mL) at 7 dpi. As in the above experiments, the protein levels of Gsdmd in the brains of the mice infected with GX074, according to Western blotting at 4 dpi, were found to be slightly lower than those in the rRC-HL and CVS-24 groups. These data indicate that pyroptosis occurred in the murine brains during the RABV infection.

### 2.5. Pyroptosis Occurred in Dissociated Neurons of Murine Brains Infected with RABV

**Figure 6:**
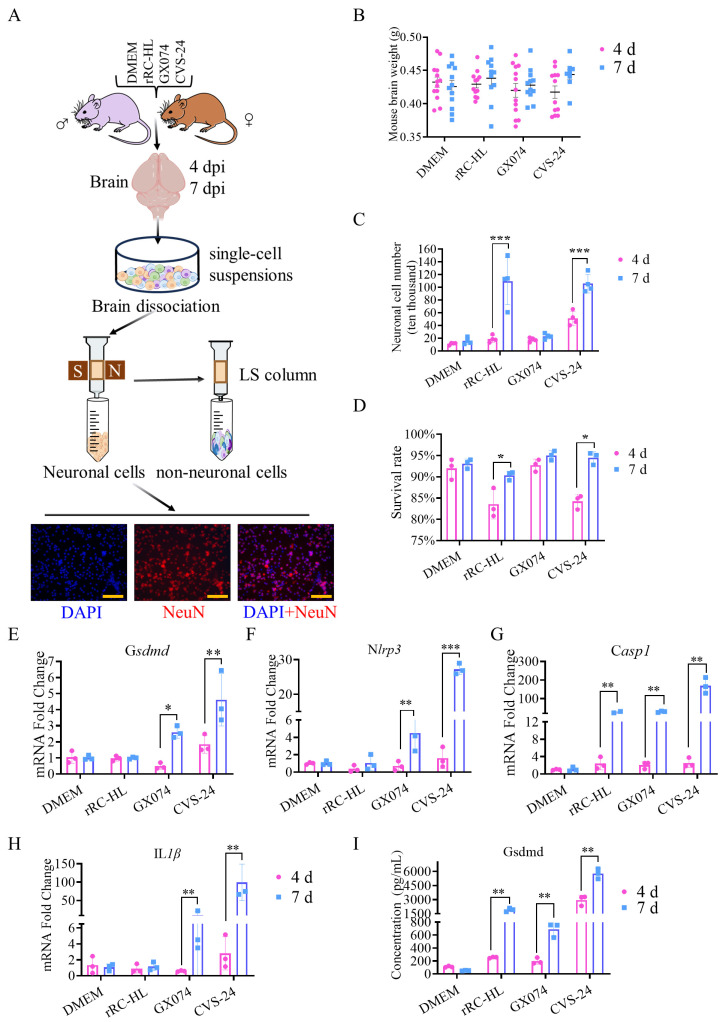
Pyroptosis occurred in dissociated neurons of murine brains infected with RABV: (**A**) The flowchart of neuronal cells’ dissociation from the brains of mice inoculated intracerebrally with DMEM, rRC-HL, GX074, and CVS-24. The purity and morphology of the dissociated neuronal cells were assessed by means of IFA (NeuN, red; DAPI, blue, Scale bar = 100 μm). (**B**) The weight of the collected murine brains (*n* = 12). (**C**) Numbers of neuronal cells isolated from the brains of mice infected with RABV. (**D**) Detection of the neuronal cell survival rate (%) was conducted with trypan blue. (**E**–**H**) Determination of pyroptosis-related factors in neuronal cells by means of RT-qPCR. (**I**) Gsdmd concentration of neuronal cells dissociated from the brains of mice infected with RABV. Asterisks indicate the statistical significance as follows: *, *p* < 0.05; **, *p* < 0.01; ***, *p* < 0.001.

To further confirm the correction between the pathological changes in neuronal cells and pyroptosis, the neuronal cells in the brains of the mice infected with RABV were dissociated using a neuron isolation kit. The flowchart of neuronal cell dissociation in the murine brains is shown in Figure 6A. Individual neuronal cells were dissociated via a specific S/N column, and individual non-neuronal cells were dissociated via the LS column. All of the separated neuronal cells were stained red with the specific anti-Neun Mab, and the nucleus was stained with DAPI. To determine whether the weight of the mice’s brains was affected by the different virulence of the RABV strains rRC-HL, GX074, and CVS-24, all of the murine brains in the experiments were weighed. As a result, we found no significant differences in the weight of the whole murine brains with or without RABV rRC-HL, GX074, or CVS-24 infection (Figure 6B). However, the total number of individual neuronal cells dissociated from each individual brain showed a certain degree of difference. Compared to the DMEM group, which had 113.31 × 10^3^ neuronal cells, the number in the rRC-HL group was 188.28 × 10^3^ cells, the GX074 group had 176.89 × 10^3^ cells, and the CVS-24 group had 511.05 × 10^3^ cells at 4 dpi; however, the neuronal cell numbers were increased at 7 dpi, where the rRC-HL group had 770.83 × 10^3^ cells, the GX074 group had 229.84 × 10^3^ cells, and the CVS-24 group had 1058.92 × 10^3^ cells. The DMEM group had 115.18 × 10^3^ cells at 7 dpi, which was similar to the number at 4 dpi. There was no significant difference between the GX074 group and the DMEM group. The greatest numbers of neuronal cells collected from individual brains were obtained from the CVS-24 group at both 4 dpi and 7 dpi (Figure 6C). 

The survival rate of the dissociated single neuronal cells was then measured using trypan blue. The results showed that the survival rate of dissociated neuronal cells from the brains of the mice infected with rRC-HL (83.53%) and CVS-24 (84.21%) at 4 dpi was slightly lower than that of those from the mice injected with DMEM (91.97%). However, the survival rate of dissociated neuronal cells from the brains of the mice infected with GX074 (92.73%) at 4 dpi did not show a significant difference. Furthermore, the rRC-HL (90.33%)-, GX074 (95.03%)-, and CVS-24 (94.47%)-infected groups did not show any significant differences compared with the DMEM group (93.1%) at 7 dpi (Figure 6D). 

Next, the mRNA expression levels of the genes related to the pyroptosis pathway from the dissociated neuronal cells were detected by means of RT-qPCR. The mRNA levels of the *Gsdmd*, *Nlrp3*, *IL-1β*, and *casp1* genes from the dissociated neuronal cells of the murine brains infected with CVS-24 increased by 4.61-, 27.25-, 169.98-, and 99.48-fold, respectively, at 7 dpi and by 2.6-, 4.52-, 28.82-, and 10.02-fold from the dissociated neuronal cells of the murine brains infected with GX074 at 7 dpi, but no change was found in the dissociated neuronal cells of the murine brains infected with rRC-HL at 4 and 7 dpi (Figure 6E–H). The Gsdmd protein is a key component in pyroptosis. The protein levels of Gsdmd from the dissociated neuronal cells infected with rRC-HL and GX074 did not significantly change at 4 dpi, but they were significantly changed by CVS-24 at 4 dpi. The mean Gsdmd concentration was determined to be 1913.8, 691.1, and 5764.9 pg/mL in the dissociated neuronal cells infected with rRC-HL, GX074, and CVS-24, respectively, at 7 dpi (Figure 6I).

## 3. Discussion

Rabies is an ancient and serious zoonosis that causes disease or death in warm-blooded mammals, including humans. The clinical manifestations of rabies patients or infected animals are remarkable, displaying distinctive neurological symptoms such as hydrophobia, fear, high excitement, and mania. Finally, almost 100% of patients and infected animals die. Based on histoanatomic observation and histopathological image analysis, no macroscopic specific pathological changes can be seen, except that a non-suppurative inflamed lesion can be found in most brain tissue samples from rabies patients or infected animals [21]. Many studies have shown that the pathological changes caused by different RABV strains are inconsistent [22,23,24,25].

The inflammatory reactions are also variable in severity (mild to severe), as is the degree of infiltration by inflammatory cells (such as mononuclear cells) [23]. Moreover, a conspicuous neuronophagia phenomenon can be seen at the histopathological level in the brains of patients or infected animals. Although many studies have explored the pathogenesis of rabies, the pathogenic mechanism by which rabies virus induces rabies and causes the death of humans and animals is still unclear. In this study, we found that many neuronal cells died as a result of RABV infection through histopathological examination. The damaged or dead neuronal cells displayed nuclear condensation, nuclear membrane rupture, and scattering of granules and vesicles around the nucleus. For this reason, we attempted to explore the mechanism of neuronal cell damage or death during RABV infection.

In the present study, the mice infected with the avirulent strain rRC-HL experienced a proliferation of glial cells but retained the integrity of their neuronal cells. Few neuronal cells died as a result of the rRC-HL infection. However, neuronal cell damage and conspicuous neuronophagia phenomenon in the neuronal cells could be seen in the hippocampus region of the brains of the mice infected with the virulent CVS-24 strain and the street GX074 infection. Necrosis was present in the fewest neuronal cells in the mice infected with GX074. Similarly, the presence of necrosis was observed in numerous cortical, hippocampal, and Purkinje neurons in the mice infected with CVS-24, whereas minimal necrosis was identified in the mice infected with silver-haired bat rabies [25]. Neuronal necrosis with satellitosis and neuronophagia was also observed in the cerebrum of camels with rabies encephalitis [26].

To find out the reasons that cause neuronal cell damage or death during RABV infection, we performed a transcriptomics analysis. According to the transcriptomics expression profiles, the brains of the mice infected with RABV showed a large number of DEGs, including pyroptosis-related factors, and cytokines, including chemokines. Our experimental results show that seven chemokines were identified as up-regulated during RABV infection by RT-qPCR and ELISA. These chemokines included CCL2, CCL3, CCL5, CCL7, CCL12, CXCL10, and CXCL11. Also, elevated concentrations of pro-inflammatory chemokines were identified in dogs and human subjects infected with street RABV strains [27]. Our previous studies have shown that microglia are involved in upregulating inflammatory cytokines and chemokines [28]. Microglia might play an important role in CNS infection, where viruses cause chemokine production that could activate microglia and thereafter induce neuroinflammation in the CNS [29].

RABV infection caused damage to or death of neuronal cells, and pyroptosis-related factors and chemokines were induced in the mice’s brains during the RABV infection. In the present experiment, the mRNA and protein levels of the key molecules Gsdmd, Nlrp3, and caspase-1 were increased, similar to those of the inflammatory factor IL-1β, which activates the pyroptosis pathway. These results are similar to a previous report of infection with the Duvenhage virus (a rabies-related virus from Africa) [4]. These data imply that RABV causing damage to or death of neuronal cells might be mediated via the pyroptosis pathway. Therefore, to further investigate whether or not RABV infection induces pyroptosis, neuronal cells were dissociated from the murine brains infected with RABV, and then the mRNA and protein expression levels of pyroptosis-related factors were measured.

As expected, the neuronal cells were successfully dissociated from the murine brains infected with RABV, and the integrity of those dissociated neuronal cells was assessed and compared with those from the brains of normal mice without RABV infection. In our experiments, mRNA changes in the pyroptosis-related genes *Gsdmd*, *Nlrp3*, *IL-1β*, and *casp1* were identified in the neuronal cells of the murine brains infected with RABV.

Pyroptosis is an important signal pathway to neuronal cell damage or death that depends on Gsdmd activation and the production of inflammatory cytokines such as caspases 1, 4, and 11 [20]. Our experiments confirmed an increase in the concentration of Gsdmd in the neuronal cells. These data suggest that pyroptosis is one of the pathogenic mechanisms of neuronal injury or death caused by rabies virus.

Pyroptosis is a type of cell death involving a strong inflammatory response that has been implicated in the development of many diseases. Previous studies showed that inhibition of the pro-inflammatory response associated with pyroptosis by inhibiting CASP-1 expression demonstrated a beneficial effect on the survival time of mice [30,31,32]. To sum up, in this study, we found that RABV infection causes pyroptosis of neuronal cells. The finding will be helpful in developing a novel strategy for rabies treatment.

## 4. Materials and Methods

### 4.1. Viruses, Animals, and Viral Infection

The RABV strains used in this study were rRC-HL, GX074, and CVS-24. The avirulent rRC-HL strain was rescued from an infectious cDNA clone pRC-HL (kindly provided by Professor Minamoto Nobuyuki, Gifu University, Gifu, Japan) based on the fixed RABV RC-HL strain used as a vaccine for animals in Japan [33]. The street virus GX074 strain was obtained from a canine brain in Guangxi Province, China. The standard challenge virus strain CVS-24 was kindly provided by Professor Ling Zhao of Huazhong Agricultural University, China. In the P3 biosafety laboratory, all virus experiments were performed in microbiological safety cabinets. 

This study was approved by the Animal Experiment Committee of Guangxi University with the approval number GXU2019-021 and conducted according to the Chinese regulations and standards for the use of laboratory animals. Healthy specific-pathogen-free (SPF) male and female Kunming mice (4 weeks old) were purchased from Guangxi Medical University, Nanning, China. After 2 days of acclimatization, the mice were randomly divided into four groups (*n* = 6) and inoculated intracerebrally (i.c.) with 30 μL of Dulbecco’s modified Eagle’s medium (DMEM, Gibco, Waltham, MA, USA) containing 10^3^ focus-forming units (FFUs) of rRC-HL, GX074, or CVS-24, while the same volume of DMEM was used as a mock group. The virus suspension was drawn into a sterile syringe (0.1 mL or 0.5 mL) connected with a segmented injection needle. The mice were inoculated intracerebrally at a point between the eye and the ear on the skull. The clinical symptoms, including body weight, were observed and recorded three times a day for 10 days post inoculation (dpi). The symptoms of the mice were scored as follows: 0, normal; 1.0, disheveled back hair, low energy, slight weight loss; 2.0, ruffled fur, decreased appetite, sensitive to stimulation; 3.0, depressed or manic behavior, irregular wandering, obviously thin; 4.0, paralysis of fore or hind limbs, unable to turn over; and 5.0, respiratory failure, death. According to previous studies in our lab, the mice showed the first clinical symptoms at 4 dpi. As the time went on, the clinical symptoms of the mice became gradually severe, and the clinical symptoms could be scored as 2.0, 3.0, 4.0, and 5.0 respectively. At 6–7 dpi, the mice presented typical neurological symptoms and were moribund, and the mice were scored as 5.0, and almost all the mice died at 6–7 days. Therefore, the mice were euthanatized with isoflurane in a closed container at 4 dpi and 7 dpi [34]. The mice’s brains were collected for further experiments.

### 4.2. Hematoxylin and Eosin (HE) Staining

Hematoxylin and eosin (HE) staining was used to prepare the tissue sections in order to observe the cellular and tissue structures of the mice’s brains in detail. The collected brain tissue was fixed in a 4% paraformaldehyde solution for over 24 h and processed using conventional paraffin embedding. The tissue sections were sliced to a thickness of 4 μm using a paraffin slicer, placed on glass slides, and baked at 60 °C before being stored at room temperature. The tissue sections were stained with hematoxylin solution for 3–5 min and eosin dye for 5 min, and then they were observed and imaged using an optical microscope (OLYMPUS, Tokyo, Japan).

### 4.3. Transmission Electron Microscope (TEM) Scanning

The murine brain tissues were cut into 1 mm^3^ cubes and fixed with 2.5% glutaraldehyde for 24 h, followed by post-fixation in 1% osmium tetroxide for 3 h. After being dehydrated in gradient ethanol of 50%, 70%, 90%, and 100% for 10 min each, the samples were embedded in resin and cut into tissue sections with a thickness of 50–60 nm. These sections were stained with uranyl acetate and lead citrate and observed under a transmission electron microscope (HT7700, Hitachi, Tokyo, Japan).

### 4.4. Bioinformatics Analysis

Transcriptome expression profile data (GSE30577) from the NCBI GEO database (http://www.ncbi.nlm.nih.gov/geo/ (accessed on 10 July 2021)) were analyzed [35]. The R package and limma were used for the analysis of differentially expressed genes (DEGs). Genes were considered to be differentially expressed if they presented with adjusted *p*-values of <0.05 and a fold change of more than 2.0 between groups (|FC| ≥ 2.0). Gene Ontology (GO, http://geneontology.org (accessed on 28 July 2021)) terms and Kyoto Encyclopedia of Genes and Genomes (KEGG, https://www.genome.jp/kegg/ (accessed on 28 July 2021)) pathway analysis were performed using homemade scripts for the R software (version R 4.0). The *p*-values were adjusted with the false discovery rate correction in the R package, and GO–BP (biological process), GO–CC (cellular component), GO–MF (molecular function), and KEGG pathway categories with adjusted *p*-values of <0.05 were defined as statistically significant.

In the functional protein association networks (STRING, http://cn.string-db.org (accessed on 23 August 2021)), we imported the list of DEG names and set the organism as “Mus musculus”, set the confidence to >0.9, and then exported the pivotal hub genes of the protein–protein interaction (PPI) results as a tabular text output, after which we imported the file into Cytoscape (version 3.8.0) for further analysis and applied the layout network. The immune-related pathways of pivotal hub genes were enriched and analyzed using the ClueGO plugin (http://apps.cytoscape.org/apps/cluego (accessed on 8 September 2021)) in the Cytoscape software.

### 4.5. Total RNA Extraction and Real-Time Quantitative PCR (RT-qPCR)

Total RNA was extracted from murine brain tissues using TRIzol Reagent (Invitrogen, Carlsbad, CA, USA) and transcribed with FastKing gDNA Dispelling RT SuperMix (TIANGEN, Beijing, China, KR118). The mRNA levels were quantified using Universal SYBR Green Supermix (ABconal, Woburn, MA, USA, RK21203) and specific primers (Table 2) on a Roche LightCycler^®^ 96 RT-qPCR instrument (Roche, Basel, Switzerland). Threshold cycle (Ct) values were normalized using β-actin as an internal control. The relative mRNA levels are presented as 2^−Δ∆Ct^ values.

### 4.6. Western Blotting

Western blotting was performed for protein detection as previously described in [36]. The mice infected with RABV at 4 dpi and 7 dpi were instantly collected and frozen in a −80 °C refrigerator for storage until use. The brain tissues were ground to a fine powder with a mortar and pestle after freezing with liquid nitrogen. The brain samples were homogenized in RIPA lysis buffer (sc-24948, Santa Cruz Biotechnology Inc., Dallas, TX, USA) containing 1% protease inhibitor and phosphatase inhibitor cocktails, and then they were further centrifuged at 12,000× *g* and 4 °C for 30 min. An equal amount of protein for each sample was loaded onto 10% SDS-PAGE gel and then electrophoresed. The proteins in the SDS-PAGE gel were transferred onto polyvinylidene difluoride (PVDF) membranes (Millipore, Billerica, MA, USA) by means of Western blotting. The PVDF membranes were blocked with 5% non-fat blocking-grade milk (Bio-Rad, Hercules, CA, USA) and then incubated overnight at 4 °C with the primary antibodies against Gsdmd (ab 219800, Abcam, Boston, MA, USA), Nlrp3 (M035175, Abmart, Shanghai, China), β-actin (anti-Actin (AA128; Beyotime, Shanghai, China), and rabies virus nucleoprotein (N) (ab-0056, Tongdian biotech, Zhejiang, China). After 5 washes with 1× TBST, the PVDF membranes were incubated with the corresponding secondary antibody at room temperature for 1 h. Chemiluminescence signals were measured using BeyoECL Plus (P0018S, Beyotime).

### 4.7. Enzyme-Linked Immunosorbent Assay (ELISA)

The ELISA kits were purchased from 4A Biotech (Suzhou, China) for the determination of CCL2, CCL3, CCL4, CCL5, CCL7, CCL12, CXCL10, and IL-1β protein concentrations. A mouse Gsdmd SimpleStep ELISA^®^ Kit (ab233627, Abcam) was used for the determination of Gsdmd levels. All kits were used according to the manufacturer’s instructions.

### 4.8. Dissociation of Neuronal Cells from Murine Brain Tissue

First, the meninges and blood vessels of the brains of mice infected with RABV were carefully removed with ophthalmic scissors at 4 dpi and/or 7 dpi. Then, the brain was cut into small pieces with a sterile sharp scalpel, after which the enzyme mixture from an Adult Brain Dissociation Kit (130-107-677, Miltenyi Biotec, Cologne, Germany) was added and incubated at 37 °C for 30 min to digest the brain into single cells. These single-cell suspensions were magnetically labeled using a Neuron Isolation Kit (130-115-389, Miltenyi Biotec) and passed through LS columns (130-042-401, Miltenyi Biotec) attached to an OctoMACS Separator (130-091-051, Miltenyi Biotec). The labeled non-neuronal cells were retained in the LS columns, while the unlabeled neuronal cells passed through and were collected.

Finally, the dissociated neuronal cells were transferred to Petri dishes, fixed with 4% formaldehyde, incubated with an anti-NeuN antibody (ab177487, Abcam) for fluorescent labeling, and photographed with an imaging system to observe the purity of the neuronal cells.

### 4.9. Statistical Analysis

Data were analyzed using the GraphPad Prism software, version 8.0 (GraphPad Software, La Jolla, CA, USA). The statistical differences between groups were analyzed using Student’s *t*-test or one-way analysis of variance. For all tests, the following notation was used to represent significant differences: * *p* < 0.05; ** *p* < 0.01; *** *p* < 0.001.

## 5. Conclusions

In this study, we found that neuronal cells were injured or died in mouse brains during RABV infection. With the help of transcriptome analysis and neuronal cell dissociation, the neuronal cells were successfully dissociated from the brains of mice infected with RABV, and we confirmed that pyroptosis occurred in the neuronal cells in which the pyroptosis-related genes *Gsdmd*, *Nlrp3*, *IL-1β*, and *caspase-1* were upregulated. These data suggest that rabies virus infection causes the pyroptosis of neuronal cells.

## Figures and Tables

**Table 1:** Differentially expressed genes (mRNA) in mouse brains infected with RABV.

ID	logFC	AveExpr	t	*p* Value	Adj. *p* Value	B
Gsdmd	2.844205356	1.388442133	21.59042636	2.69 × 10^−11^	1.14 × 10^−7^	16.17239136
Il1b	3.113935602	1.615343525	8.434830498	1.60 × 10^−6^	7.98 × 10^−5^	5.460571846
Il18bp	3.603517781	1.814911737	10.25283251	1.85 × 10^−7^	1.57 × 10^−5^	7.66599915
Il18r1	2.389778263	1.387312137	3.990398537	0.001647569	0.01953493	−0.672939539
Casp1	2.340518378	1.107677817	11.50754131	4.96 × 10^−8^	6.26 × 10^−6^	8.998377851
Casp4	4.15842864	2.205378074	6.956852555	1.21 × 10^−5^	0.000390382	3.381602478
Casp8	2.131884685	1.184125641	5.892775917	6.13 × 10^−5^	0.001403526	1.70297839
Myd88	2.196086505	1.064698849	13.3000403	9.18 × 10^−9^	1.95 × 10^−6^	10.68070028
Ccl2	6.24356608	3.411944274	6.330990977	3.08 × 10^−5^	0.000818871	2.413486338
Ccl3	4.674781418	2.386502145	6.971375474	1.18 × 10^−5^	0.000383974	3.403424777
Ccl4	4.8957759	2.612624677	7.659116565	4.47 × 10^−6^	0.000175587	4.404433247
Ccl5	5.622229301	3.014193216	6.583590985	2.10 × 10^−5^	0.000604757	2.810778671
Ccl6	2.074863529	1.005737009	9.688097084	3.50 × 10^−7^	2.53 × 10^−5^	7.018317637
Ccl7	4.630499516	2.578568689	6.17806434	3.90 × 10^−5^	0.000980812	2.168607594
Ccl8	3.923844391	2.271585036	5.066016644	0.000241605	0.004282622	0.289894715
Ccl11	2.523599811	1.33807791	5.583445545	0.000101271	0.00211264	1.185215262
Ccl12	4.053627344	2.193295352	7.435890891	6.09 × 10^−6^	0.000223045	4.086394997
Ccl19	3.047127763	1.57842593	11.37181271	5.69 × 10^−8^	6.90 × 10^−6^	8.860871153
Cxcl1	2.912314782	1.702943343	4.921608719	0.000309992	0.00527857	0.033776361
Cxcl5	2.337632723	1.115456774	7.991835275	2.86 × 10^−6^	0.000125389	4.866589008
Cxcl9	7.503478056	4.346227113	6.947762239	1.22 × 10^−5^	0.00039427	3.367928611
Cxcl10	6.165935098	3.59249242	5.575971475	0.000102524	0.002134122	1.172540508
Cxcl11	5.406051233	3.134538595	6.574184691	2.13 × 10^−5^	0.000610855	2.796144328
Cxcl13	4.956874491	2.647619269	10.97854878	8.52 × 10^−8^	8.89 × 10^−6^	8.453647681
Cxcl16	2.694888761	1.4887887	9.960898774	2.57 × 10^−7^	2.00 × 10^−5^	7.33514306
Cxcr3	2.471460721	1.245877913	8.936492225	8.55 × 10^−7^	4.93 × 10^−5^	6.105091257
Cxcr4	2.249708912	1.243362164	5.491198936	0.000117929	0.002380542	1.028242724
Cxcr6	3.143133937	1.77411466	5.055090275	0.00024618	0.004349175	0.270607964
Ccr1	2.37645599	1.222525505	9.203672535	6.19 × 10^−7^	3.85 × 10^−5^	6.436710981
Ccr2	3.144175717	1.63333426	8.288871024	1.93 × 10^−6^	9.26 × 10^−5^	5.267499191
Ccr5	2.984380392	1.548023314	6.771390822	1.58 × 10^−5^	0.000483111	3.100383633
Ccr7	3.18192383	1.481011385	9.838776405	2.95 × 10^−7^	2.18 × 10^−5^	7.194242366
Ccrl2	2.499253762	1.356145426	4.766588932	0.000406348	0.006472255	−0.244013599
B2m	2.770761	1.407537	15.26963608	1.78 × 10^−9^	6.80 × 10^−7^	12.28174
Tnf	4.052193	1.972883	19.05945276	1.23 × 10^−10^	2.22 × 10^−7^	14.80289
Il6	5.613003	2.905548	11.21191994	6.69 × 10^−8^	7.67 × 10^−6^	8.696901
Il10	2.437385	1.177951	7.129583998	9.40 × 10^−6^	0.000317	3.639293
Stat1	2.804149	1.350931	15.7930469	1.19 × 10^−9^	5.39 × 10^−7^	12.67005
H2-D1	2.800854	1.336032	23.05218819	1.21 × 10^−11^	1.05 × 10^−7^	16.8725
H2-K1	3.281039	1.649339	13.68077695	6.58 × 10^−9^	1.56 × 10^−6^	11.00877
H2-T23	3.143764	1.577127	15.74253308	1.24 × 10^−9^	5.39 × 10^−7^	12.6332

**Table 2:** Primers used for detection of chemokines and pyroptosis-related factors by RT-qPCR.

Gene Name	Primer Sequences (5′ to 3′)
*Ccl2*-F	CAGCCAGATGCAATCAATGC
*Ccl2*-R	GAATCCTGAACCCACTTCTG
*Ccl3*-F	CCTTGCTGTTCTTCTCTGTACC
*Ccl3*-R	TCAGTGATGTATTCTTGGACCC
*Ccl4*-F	TTCCTGCTGTTTCTCTTACACCT
*Ccl4*-R	CTGTCTGCCTCTTTTGGTCAG
*Ccl5*-F	ATGAAGATCTCTGCAGCTGC
*Ccl5*-R	CACTTGCTGCTGGTGTAGAA
*Ccl7*-F	GCTGCTTTCAGCATCCAAGTG
*Ccl7*-R	CCAGGGACACCGACTACTG
*Ccl12*-F	ATTTCCACACTTCTATGCCTCCT
*Ccl12*-R	ATCCAGTATGGTCCTGAAGATCA
*Cxcl10*-F	CCAAGTGCTGCCGTCATTTTC
*Cxcl10*-R	GGCTCGCAGGGATGATTTCAA
*Gsdmd*-F	CCATCGGCCTTTGAGAAAGTG
*Gsdmd*-R	ACACATGAATAACGGGGTTTCC
*Nlrp3*-F	ATTACCCGCCCGAGAAAGG
*Nlrp3*-R	TCGCAGCAAAGATCCACACAG
*Casp1*-F	ACAAGGCACGGGACCTATG
*Casp11*-R	TCCCAGTCAGTCCTGGAAATG
*IL-1β*-F	GCAACTGTTCCTGAACTCAACT
*IL-1β*-R	ATCTTTTGGGGTCCGTCAACT
*β-actin*-F	AGACCTCTATGCCAACACAGT
*β-actin*-R	CATCGTACTCCTGCTTGCTGAT

## Data Availability

Data is contained within the article.
